# Clinical and laboratory factors associated with mortality among hospitalized patients with COVID-19 infection in Lebanon: A multicenter study

**DOI:** 10.1371/journal.pone.0278393

**Published:** 2022-12-01

**Authors:** Marianne Chebli, Anthony Shebly, Georges Kerbage, Christian Joseph El Zouki, Elissar Hayek, Pascale Salameh, Rabih Hallit, Souheil Hallit

**Affiliations:** 1 School of Medicine and Medical Sciences, Holy Spirit University of Kaslik, Jounieh, Lebanon; 2 School of Medicine, Lebanese American University, Byblos, Lebanon; 3 Institut National de Santé Publique, Epidémiologie Clinique et Toxicologie (INSPECT-LB), Beirut, Lebanon; 4 Department of Primary Care and Population Health, University of Nicosia Medical School, Nicosia, Cyprus; 5 Faculty of Pharmacy, Lebanese University, Hadat, Lebanon; 6 Infectious Disease Department, Notre Dame des Secours University Hospital Center, Byblos, Lebanon; 7 Infectious Disease Department, Bellevue Medical Center, Mansourieh, Lebanon; 8 Research Department, Psychiatric Hospital of the Cross, Jal Eddib, Lebanon; 9 Applied Science Research Center, Applied Science Private University, Amman, Jordan; Stanford University School of Medicine, UNITED STATES

## Abstract

**Background:**

With the dire economic situation in Lebanon, many of the basic resources of the hospitals needed to help fight COVID-19 infections are not available. In this paper, we studied the possible factors associated with increased mortality in a sample of Lebanese adults enrolled in three hospitals.

**Method:**

In this retrospective cohort study, we analyzed data from 416 adults hospitalized in three institutions for a COVID-19 infection, from the opening of the COVID unit until their closure (period extending from March 2020 to June 2021). We used multivariate analyses to assess potential factors associated with COVID-19 mortality: gender, age, the presence of underlying medical conditions, and some medication taken during hospitalization.

**Results:**

Using variables related to baseline characteristics entered as independent variables, acute kidney injury (aOR = 4.057) and older age (aOR = 1.053) were associated with a higher probability of death. After adjusting baseline characteristics and factors related to admission entered as independent variables, enoxaparin intake (aOR = 0.435) was significantly associated with a lower probability of death, whereas old age (aOR = 1.049) and ventilation (aOR = 1.2) were significantly associated with higher odds of death. When all variables that showed significance in bivariate analysis were entered, old age (aOR = 1.243) and highest PaCO2 during hospitalization (aOR = 1.192) were significantly associated with higher mortality. With a weak effect, atrial fibrillation, COPD, and higher leucocyte counts on admission were significantly associated with higher odds of death.

**Conclusion:**

These findings could help us prevent severe diseases in patients with several comorbidities and adjust therapeutic care to improve future outcomes. More studies should compare the outcome of different COVID-19 strains as well as the impact of vaccination on those with multiple comorbidities, especially on the mortality rate culminating from disease complications.

## Introduction

Since 2019, the Severe Acute Respiratory Syndrome-CoronaVirus-2 (SARS-CoV-2) pandemic has contributed to many deaths worldwide. Originating in Wuhan, China, at the end of September 2022, it has rapidly spread globally, leading to nearly 6.54 million deaths and 622 million confirmed cases by September 30, 2022 [1]. By that time, the total number of confirmed cases in Lebanon had reached 1.2 million and 10,674 deaths [1].

Many variants have been identified by 2022, and WHO divided the variants into variants of concern (VOC), variants of interest (VOI), variants being monitored (VBM), and variants of high consequences (VOHC). Some of these variants include B.1.1.7, also known as Alpha, B.1.617.2, also called Delta, and B.1.1.529, or Omicron. Up to May 2022, Alfa and Delta were classified as VBM in the US, and omicron is still a VOC [2].

The presentation of the disease varies from fever, cough, and mild respiratory symptoms to more severe symptoms (hypoxia, respiratory failure) [3], that can subsequently lead to more fatal complications (multiple organ failure, septic shock, pulmonary edema, severe pneumonia, acute respiratory distress syndrome (ARDS), and death) [4–6]. Such clinical presentation may require the physician to admit the patient to the hospital or intensive care unit (ICU) [7,8]. Those severe complications can be explained by the fact that SARS-CoV-2 is thought to trigger a cytokine storm, which activates a severe inflammatory response in the body, leading to those fatal complications [9–11]. Healthcare professionals have been through many daily challenges while facing this pandemic, since the clinical presentation can differ from one patient to another, and the management of COVID-19 was not fully standardized and clear at the time, knowing that some patients with mild symptoms can deteriorate rapidly after hospital admission [11–13]. It has been proven that SARS-CoV-2 is more virulent and aggressive in elderly patients and those with common comorbidities such as hypertension, diabetes, coronary heart disease, obesity, COPD, cancer, chronic kidney disease, and hemodialysis. [14–19]. On the other hand, many treatments were used in order to decrease the severity of COVID-19 infection. One of the widely used treatments is enoxaparin. Several studies showed that enoxaparin could be a protective factor in the course of a COVID-19 infection ([20–22]).

Due to the dire economic situation in Lebanon, a small country in the Middle East, the government has been unable to deliver a stimulus package to provide public and private hospitals with much-needed resources to fight the disease. Therefore, the basic financial resources required by hospitals to help patients in need are becoming very limited. In order to fight the pandemic, public hospitals relied almost entirely on WHO, as well as foreign and local non-governmental assistance to import essential diagnostic kits, personal protective equipment (PPE), and other required materials and equipment [23,24]. Because of this, we emphasize the fact that patients’ profiles should be identified and evaluated as soon as they become infected with COVID-19. This will allow clinicians and hospitals to allocate resources appropriately and focus on efficient medical interventions that could potentially decrease morbidity and death.

To the best of our knowledge, no studies identified the link between clinical and laboratory findings among hospitalized patients with COVID-19 in the Lebanese setting. Therefore, our objectives in this study are to identify clinical and laboratory factors associated with increased mortality among hospitalized patients with COVID-19 infection.

## Methods

### Ethics approval and consent to participate

The study protocol was approved by the ethics committee of the Notre-Dame des Secours University Hospital and Bellevue Medical Center. The approval done by Notre-Dame des Secours University Hospital ethics committee was also used in Bhannes Medical Center. Each patient was contacted to get his written informed consent prior to enrolling him/her in the study. Consent was obtained from immediate family members in case of death.

### Patients and data collection

This study is a retrospective cohort study on 416 enrolled adults with PCR-confirmed COVID-19 who were consecutively admitted to the three institutions for a COVID-19 infection, from the opening of the COVID units until their closure (period extending from March 2020 to June 2021). The COVID units included a COVID floor and an ICU. Data on COVID-19 cases and deaths were extracted from the computerized system or charts according to the availability of the information. Patients with incomplete information, patients younger than 18 years, and patients with a negative COVID-19 RT-PCR or an unverified COVID-19 result were excluded. Patients were classified into (0) asymptomatic, (1) mild, (2) moderate, or (3) severe disease depending upon their symptoms at presentation. Asymptomatic patients or people without any reported clinical symptoms were hospitalized at the beginning of the pandemic in Lebanon to decrease COVD-19 fear and to decrease the disease contagion. Mild symptoms include patients with a documented COVID-19 infection without the suggestion of viral pneumonia or hypoxia. Patients with moderate disease of pneumonia include patients with no evidence of severe pneumonia, and SpO2≥90%; Severe and critically ill patients include patients with severe pneumonia,SpO2<90%, acute respiratory distress syndrome (ARDS), sepsis, and septic choc [25]. Data collection was done according to the sheet found in S1 Appendix.

### Statistical analysis

The SPSS software v.22 was used for the statistical analysis. The Chi-square test was used to compare two categorical variables, whereas the Student t test was used to compare two means. A logistic regression was then conducted, taking mortality in the COVID-19 unit as the dependent variable. All variables that showed a p<0.25 in the bivariate analysis were entered as independent variables. Significance was set at p<0.05.

## Results

### Sociodemographic characteristics

A total of 415 patients accepted to participate in the study, with a mean age of 61.58 ± 16.34 years and 32.0% females; 93 (22.4%) patients died. Other characteristics of the sample are displayed in Table 1.

**Table 1:** Sociodemographic characteristics of the patients (N = 415).

**Variable**	**N (%)**
**Gender**	
Male	282 (68.0%)
Female	133 (32.0%)
**Admission to ICU**	
No	293 (70.6%)
Yes	122 (29.4%)
**Grading**	
Asymptomatic	12 (2.9%)
Mild	76 (18.4%)
Moderate	202 (48.8%)
Severe	124 (30.0%)
**Death**	
No	322 (77.6%)
Yes	93 (22.4%)
	**Mean ± SD**
Age (in years)	61.58 ± 16.34
Body Mass Index (kg/m^2^)	28.31 ± 4.98

### Bivariate analysis

A significantly higher percentage of males, of those who were admitted to the ICU, of those who had severe COVID-19 infection, who had hypertension, congestive heart failure, atrial fibrillation, dementia, peripheral vascular or liver disease, who were oxygen-dependent at home due to other diseases, who had thrombotic complications during hospitalizations, endotracheal intubation during hospitalizations, dialysis during hospitalization, who were vented, and were not treated with enoxaparin at their admission day as a prophylactic or therapeutic measure. Moreover, a higher mean age, leukocyte count, CRP upon admission, total length of hospital stay, vented length of stay, and PCO2 worst value were significantly associated with higher odds of death; furthermore, a lower mean platelet count, D-dimer count, PO2 upon admission, PO2 worst value, and pH upon admission were also associated with higher mortality (Table 2).

**Table 2:** Bivariate analysis of death as the dependent variable.

	Death		p-value
	No (N = 321)	Yes (N = 92)	
**Gender**			
Male	225 (79.8%)	57 (73.2%)	0.131
Female	97 (72.9%)	36 (27.1%)	
**Admission to ICU**			
No	272 (92.8%)	21 (7.2%)	**<0.001**
Yes	50 (41.0%)	72 (59.0%)	
**Grading** Asymptomatic Mild Moderate Severe	12 (100.0%) 74 (97.4%) 192 (95.0%) 44 (35.5%)	0 (0.0%) 2 (2.6%) 10 (5.0%) 80 (64.5%)	**<0.001**
Smoking status No Yes Previous smoking	227 (79.4%) 71 (76.3%) 24 (66.7%)	59 (20.6%) 22 (23.7%) 12 (33.3%)	0.239
Hypertension No Yes	169 (83.7%) 153 (71.8%)	33 (16.3%) 60 (28.2%)	**0.005**
Congestive heart failure No Yes	293 (79.2%) 29 (64.4%)	77 (20.8%) 16 (35.6%)	**0.036**
Cardiomyopathy No Yes	318 (77.6%) 4 (80.0%)	92 (22.4%) 1 (20.0%)	1
Atrial fibrillation No Yes	303 (78.9%) 19 (61.3%)	81 (21.1%) 12 (38.7%)	**0.041**
Acute Kidney Injury No Yes	317 (78.5%) 5 (45.5%)	87 (21.5%) 6 (54.5%)	**0.019**
Chronic Obstructive Pulmonary Disease No Yes	310 (78.9%) 12 (54.5%)	83 (21.1%) 10 (45.5%)	**0.015**
Oxygen-dependent at home No Yes	319 (78.8%) 3 (30.0%)	86 (21.2%) 7 (70.0%)	**0.002**
Active cancer No Yes	279 (78.6%) 43 (71.7%)	76 (21.4%) 17 (28.3%)	0.243
Seizure No Yes	310 (78.3%) 12 (63.2%)	86 (21.7%) 7 (36.8%)	0.155
Dementia No Yes	320 (78.6%) 2 (25.0%)	87 (21.4%) 6 (75.0%)	**0.002**
Peripheral vascular disease No Yes	314 (79.1%) 8 (44.4%)	83 (20.9%) 10 (55.6%)	**0.002**
Liver disease No Yes	317 (78.3%) 5 (50.0%)	88 (21.7%) 5 (50.0%)	**0.049**
Cough on admission No Yes	122 (74.4%) 200 (79.7%)	42 (25.6%) 51 (20.3%)	0.229
Dyspnea on admission No Yes	66 (84.6%) 256 (76.0%)	12 (15.4%) 81 (24.0%)	0.131
Abdominal pain on admission No Yes	299 (76.7%) 22 (91.7%)	91 (23.3%) 2 (8.3%)	0.128
Nausea on admission No Yes	301 (76.6%) 20 (100.0%)	92 (23.4%) 0 (0.0%)	**0.010**
Vomiting on admission No Yes	304 (76.8%) 17 (94.4%)	92 (23.2%) 1 (5.6%)	0.089
Olfactory or Gustatory dysfunction on admission No Yes	44 (86.3%) 76 (95.0%)	7 (13.7%) 4 (5.0%)	0.107
Headache on admission No Yes	286 (76.5%) 36 (87.8%)	88 (23.5%) 5 (12.2%)	0.116
Myalgia on admission No Yes	265 (75.5%) 56 (88.9%)	86 (24.5%) 7 (11.1%)	**0.021**
Thrombotic complications during hospitalization No Yes	318 (80.7%) 4 (19.0%)	76 (19.3%) 17 (81.0%)	**<0.001**
Endotracheal intubation during hospitalization No Yes	314 (91.8%) 8 (11.0%)	28 (8.2%) 65 (89.0%)	**<0.001**
Required dialysis during hospitalization No Yes	311 (78.9%) 11 (52.4%)	83 (21.1%) 10 (47.6%)	**0.012**
Vented No Yes	53 (100.0%) 269 (74.3%)	0 (0.0%) 93 (25.7%)	**<0.001**
Beta-blockers No Yes	228 (80.0%) 94 (72.3%)	57 (20.0%) 36 (27.7%)	0.099
Ezetimibe No Yes	313 (77.1%) 9 (100.0%)	93 (22.9%) 0 (0.0%)	0.218
Clopidogrel No Yes	292 (78.9%) 30 (66.7%)	78 (21.1%) 15 (33.3%)	0.086
Enoxaparin No Yes	35 (64.8%) 287 (79.5%)	19 (35.2%) 74 (20.5%)	**0.022**
Remdesivir No Yes	264 (76.7%) 58 (81.7%)	80 (23.3%) 13 (18.3%)	0.363
**Age**	58.76 ± 15.71	71.38 ± 14.67	**<0.001**
**Leukocyte count on admission**	8127.79 ± 4033.36	11090.12 ± 6665.33	**0.048**
**Platelet count on admission**	235090 ± 111752	228447 ±145067	**0.002**
**Creatinine on admission**	2.56 ± 23.42	1.57 ± 1.80	0.073
**Procalcitonin**	0.41 ± 1.28	0.36 ± 0.40	0.193
**Interleukin-6**	34.60 ± 24.57	113.34 ± 112.56	0.062
**CRP on admission**	101.07 ± 88.00	122.99 ± 92.71	**0.010**
**D-dimer**	163.90 ± 527.16	38.66 ± 167.35	**0.003**
**Bicarbonate**	25.43 ± 5.00	23.50 ± 5.32	0.079
**Total length of hospital stay**	8.42 ± 6.70	12.42 ± 8.64	**<0.001**
**Vented Length of Stay**	8.22 ±7.23	12.32 ± 8.97	**<0.001**
**PO2 on admission**	74.49 ± 32.83	63.62 ± 26.65	**0.046**
**PO2 worst value**	60.24 ± 18.35	43.12 ± 12.29	**<0.001**
**pH on admission**	7.45 ± 0.10	7.37 ± 0.16	**0.002**
**PCO2 on admission**	35.82 ± 11.93	41.38 ± 20.21	0.080
**PCO2 worst value**	43.85 ± 20.95	75.80 ± 32.79	**<0.001**

Numbers in bold indicate significant p-values.

### Multivariable analysis

The results of a first model, taking variables related to baseline characteristics entered as independent variables, showed that having acute kidney injury (aOR = 4.057) and older age (aOR = 1.053) were significantly associated with higher odds of death (Table 3, Model 1).

**Table 3:** Multivariable analysis: Logistic regression taking the presence vs absence of death as the dependent variable (N = 316).

Variable	p-value	aOR	95% Confidence Interval	
			Lower Bound	Upper Bound
**Model 1: Variables related to baseline characteristics entered as independent variables.**				
Gender (Female vs male*)	0.318	1.337	0.756	2.364
Smoking status Non-smoker Previous smoker Actual smoker	0.267 0.247 0.179	1 1.689 1.555	0.696 0.816	4.101 2.963
Hypertension (yes vs no*)	0.900	1.037	0.586	1.835
Congestive heart failure (yes vs no*)	0.392	1.394	0.652	2.980
Atrial fibrillation (yes vs no*)	0.688	1.213	0.474	3.104
Acute kidney injury (yes vs no*)	**0.048**	4.057	1.010	16.302
COPD (yes vs no*)	0.345	1.625	0.593	4.455
Active cancer (yes vs no*)	0.537	1.251	0.614	2.548
Age	**<0.001**	1.053	1.031	1.075
**Model 2: Baselines characteristics and factors related to the hospital admission entered as** **independent variables (N = 316).**				
Gender (Female vs male*)	0.159	1.586	0.834	3.014
Smoking status Non-smoker Previous smoker Actual smoker	0.348 0.075	1 1.611 2.027	0.595 0.931	4.363 4.412
Hypertension (yes vs no*)	0.636	1.168	0.614	2.225
Congestive heart failure (yes vs no*)	0.426	1.421	0.599	3.371
Atrial fibrillation (yes vs no*)	0.833	0.894	0.316	2.527
Acute kidney injury (yes vs no*)	0.436	1.966	0.359	10.770
COPD (yes vs no*)	0.131	2.518	0.759	8.348
Active cancer (yes vs no*)	0.123	1.901	0.840	4.304
Cough on admission (yes vs no*)	0.993	0.997	0.522	1.904
Dyspnea on admission (yes vs no*)	0.920	1.047	0.423	2.592
Beta-blockers (yes vs no*)	0.057	1.834	0.982	3.428
Enoxaparin intake (yes vs no*)	**0.039**	0.435	0.197	0.958
Age	**<0.001**	1.049	1.023	1.074
CRP on admission	0.248	1.002	0.999	1.005
Total length of hospital stay	0.149	0.904	0.788	1.037
Vented length of stay	**0.011**	1.2	1.043	1.382

Variables entered in the model: Gender, smoking status, hypertension, congestive heart failure, atrial fibrillation, acute kidney injury, Chronic Obstructive Pulmonary Disease, active cancer, cough on admission, dyspnea on admission, beta-blockers, enoxaparin, age, C-reactive protein on admission, total length of hospital stay, vented length of stay

*Reference group; aOR = adjusted odds ratio; Numbers in bold indicate significant p-values.

In the second model, taking baselines characteristics and factors related to the hospital admission entered as independent variables, the results showed that enoxaparin intake (aOR = 0.435) was significantly associated with lower odds of death, whereas older age (aOR = 1.049) and ventilation (aOR = 1.2) were significantly associated with higher odds of death (Table 3, Model 2).

When entering all the variables that showed significance in the bivariate analysis in a third model, the results showed that older age (aOR = 1.243) and a higher PCO2 worst value during hospitalization (aOR = 1.192) were significantly associated with higher odds of death (Table 4). Atrial fibrillation, having COPD and higher leukocyte count on admission were significantly associated with higher odds of death, but this effect size was very weak.

**Table 4:** Multivariable analysis: Logistic regression taking the presence vs absence of death as the dependent variable and all variables that showed significance in the bivariate analysis as independent ones (N = 88).

Variable	p-value	aOR	95% Confidence Interval	
			Lower Bound	Upper Bound
ICU admission (yes vs no*)	.055	102.751	.907	11640.225
Congestive heart failure (yes vs no*)	.204	16.905	.216	1324.654
Atrial fibrillation (yes vs no*)	**.016**	.000	.000	.147
Acute kidney injury (yes vs no*)	.864	.698	.012	42.085
COPD (yes vs no*)	**.015**	.000	.000	.149
Dementia (yes vs no*)	.558	.170	.000	63.741
Myalgia on admission (yes vs no*)	.050	.008	.000	1.009
Required dialysis upon admission (yes vs no*)	.228	.015	.000	13.922
Enoxaparin intake (yes vs no*)	.096	.023	.000	1.948
Age	**.013**	1.243	1.048	1.475
Leukocyte count on admission	**.043**	1.001	1.000	1.001
Platelet count on admission	.456	1.000	1.000	1.000
Total length of stay in the hospital	.697	.823	.309	2.194
Vented length of stay	.702	.821	.300	2.250
PO2 on admission	.063	.933	.867	1.004
pH on admission	.359	.002	.000	968.040
PCO2 worst value during hospitalization	**.002**	1.192	1.067	1.332

## Discussion

### Enoxaparin use and mortality

Although our paper did not differentiate between therapeutic and prophylactic enoxaparin dosages, analytical studies demonstrated a protective relation between its use and mortality in hospitalized COVID-19-infected patients [20,21,26]. In addition, all patients included in our study benefited from anticoagulation administration from their day of admission. Since early anticoagulation was found to be beneficial in reducing mortality in COVID-19 infections [26], this can be thought to have had a positive impact on patients’ chances of survival. In the bivariate analysis, the increase in D-dimer level on admission was clinically significant, however, in the multivariable model no major association was found. Perhaps this statistical difference could be explained by the early administration of enoxaparin and its beneficial effects on decreasing thrombus formation. The D-dimer level was lower in the mortality group; this might be explained by a difference in enoxaparin dosages, not included in our study. Depending on the initial presentation, patients had either a therapeutic or prophylactic enoxaparin dosage. Patients with a higher d-dimer count probably took a therapeutic dose that is associated with lower thromboembolism and mortality rates [27,28].

### Age and mortality

Our findings reveal that being of older age is associated with a greater chance of mortality. This isn’t surprising knowing that it’s widely accepted that advanced age is a major risk factor for COVID mortality [29,30]. A meta-analysis that included more than half a million participants from many countries worldwide highlighted the significant effect of age on COVID mortality with people over 60 years old being the most vulnerable to the virus [30].

### Acute kidney injury and mortality

Our results showed that patients who presented with Acute Kidney Injury displayed an increased likelihood of death. A retrospective cohort study revealed that people with Covid-19 frequently face complications like AKI. It has been linked to a bad prognosis and develops early along with respiratory failure [31]. Furthermore, it was also found that the primary outcome of patients with both COVID-19 and AKI was in-hospital death, and if the patient survived, dialysis to restore kidney function was imperative[32]. The positive association between AKI and older age is also supported by other studies that linked those previous factors to a higher risk of death [33,34]. All the patients in our study who came with AKI to the ER and died had severe disease at presentation (grade 3). This can be thought to be related to a delayed presentation. In fact, taking into account that Lebanon has been through a severe economic crisis [35], many citizens might have delayed their hospital admission and self-medicated due to the lack of financial resources and the inability of a deteriorating healthcare system to cover their needs. In addition, at that time, hospitals were overloaded which further delayed hospital admission for some. Therefore, it can be thought that higher mortality rates in patients with AKI, a proven complication, could be explained by their delayed presentation in the disease course.

### Atrial fibrillation and mortality

Having atrial fibrillation was also correlated with higher odds of death, however, this outcome extent was very weak, which could be due to our small sample size. Nonetheless, this effect was also seen in other studies [36]. Atrial fibrillation is a condition that increases the risk of thromboembolic events which may increase the risk of death. It has been shown that the risk of developing a new-onset AF is increased and that an aggravation of pre-existing AF is probable during the acute phase of COVID-19 infection [37]. This aggravation can be due to several mechanism including direct myocardial damage, electrolytes imbalance and a cytokine storm -through activation of the system inflammatory system- which have profound cardiovascular consequences on patients mainly in the form of AF [38]. It’s worth noting that Atrial Fibrillation is not specific to COVID-19 but is rather a widespread reaction to the systemic inflammation caused by viral illnesses such as Influenza [2].

### PaCO2 levels and mortality

Furthermore, and in synergism with other studies [39], a higher PaCO2 worst value during hospitalization showed to be associated with higher death rates. Even though other ABGs components (i.e. PaO2, pH) did not display any statistical difference in our study, Gupta and Al [39] showed a significant association between PaO2 and poorer outcomes. In addition, it has been shown that hypercapnia is common in invasively ventilated COVID-19 patients [40]. That same study also revealed that the main differences between hypercapnic and normocapnic patients were that people with hypercapnia had greater severity of ARDS, higher incidence of venous thromboembolic events, and increased ventilation rates. However, no association with mortality was proven [40].

### Ventilation duration and mortality

While our study did not differentiate between the type of ventilation used, to our knowledge, few papers studied the association between length of ventilation (invasive or noninvasive) and death rates. Our research demonstrated that a higher ventilation duration was associated with a higher death rate. This may be related to the fact that an increase in ventilation rate or its duration was associated with a higher PCO2 and an increase in ARDS severity [40]. Likewise, another study found that patients with COVID who needed ventilation had a certain degree of lung damage that was linked to a greater mortality rate [41].

### COPD and mortality

Having a history of COPD was associated with higher odds of death. Nevertheless, this effect was also seen in other studies[15]. COPD, especially those with CO2 retention, is associated with a higher PaCO2 rate. Since a higher PaCO2 was clinically significant in our study in increasing mortality rates, COPD and PaCO2 may be linked together.

### Leukocyte count and mortality

A study done by Blomme S. and Al, [42], showed that the deceased COVID-19 group had significantly higher total leucocyte counts in the first 20 days after admission compared to the recovered COVID-19 group, telling that higher degrees of inflammation lead to a more severe outcome. In our study, we found a similar but weak effect because of the small sample size. Higher leucocyte counts on admission showed a statistical diference in patients’ prognosis. This can suggest an increase in inflammation even at presentation could be linked to a poorer outcome.

### Limitations

Our limitations are mainly related to retrospective cohort limitations; the absence of some laboratory data for some patients (i.e. levels of procalcitonin, D-dimer, IL-6, ferritin, ABGs…), which may have affected their analysis in the multivariate analysis. Also, the lack of standardized care in the early COVID-19 pandemic may have affected our study since the three centers had different approaches to COVID-19 management. A specific study that allows considering management discrepancies would be necessary to confirm our findings. The number of patients who had a moderate/severe condition was 326; however, only 122 patients were admitted to the ICU, which might be a source of bias in terms of mortality. In fact, the lack of hospital and ICU beds for proper care may have affected our study, since many patients were ventilated at home before admission, and many self-medicated themselves prior to admission. Furthermore, the study’s timeline did not include vaccinated patients and newer strains (i.e. Delta, Omicron), which can have a different outcome. In addition, not differentiating between enoxaparin dosages is one of the limitations of our study. Further studies are recommended to confirm our findings.

## Conclusion

To conclude, patients with COVID-19 who benefited from enoxaparin had lower mortality rates. Older and vented patients, increased ventilation duration, patients presenting with acute kidney injury, and higher worst PaCO2 were associated with a higher mortality rate. These findings could help us prevent severe diseases in patients with several comorbidities and adjust therapeutic care to improve future outcomes. More studies should compare the outcome of different COVID-19 strains as well as the impact of vaccination on those with multiple comorbidities especially on the mortalities culminating from disease complications.

## Supporting information

S1 Appendix Data collection sheet. (DOCX)
