# Supplementary material for: Clinical and laboratory factors associated with mortality among hospitalized patients with COVID-19 infection in Lebanon: A multicenter study
Source: PLoS One. 2022 Dec 1;17(12):e0278393. doi: 10.1371/journal.pone.0278393 (PMC9714727; doi:10.1371/journal.pone.0278393)
Supplement: S1 Appendix — (DOCX) [file pone.0278393.s001.docx]

**Appendix 1. Data collection sheet**

Age:

Gender:

Height (in meters):

Weight (in Kg):

Admission to ICU: Yes/ No

Grading of COVID-19 infection:

Mortality: Yes/No If yes, how many days post admission:

Hypertension:

Dyslipidemia:

Coronary Artery Disease:

Congestive Heart Failure:

Cardiomyopathy:

Atrial Fibrillation:

Asthma:

COPD:

Oxygen dependent at home:

Chronic kidney disease:

End-stage renal disease:

Cerebrovascular accident:

Transient ischemic attack:

Peripheral vascular disease:

Diabetes mellitus:

Hypothyroidism:

Seizures:

Dementia:

Anemia:

Active cancer:

Acute kidney injury:

Anticoagulation:

Autoimmune disease:

Liver disease:

Smoking status: non-smoker / former smoker / active smoker

Number of cigarettes per day:

Number of waterpipes per week:

*Initial presenting symptoms in the emergency department:*

Cough

Fever

Dyspnea

Chest pain

Diarrhea

Abdominal pain

Loss of taste

Loss of smell

Sore throat

Nausea

Vomiting

Headache

Myalgia

Arthralgia

Fatigue

Rash

*Initial reported labs:*

Hemogobin

WBC

Lymphocytes

Neutrophils

Platelets

BUN

Creatinine

Albumin

Ferritin

Procalcitonin

IL-6

CRP

D-dimer

Lactic acid

LDH

Fibrinogen

Bicarbonate

Total length of stay in the hospital (in days)

ICU stay

ICU length of stay

Proning

Number of proning days

Required dialysis inpatient

Vented

Vented LOS

Stroke

Readmission within 7 days

Readmission within 30 days

Oxygen (defined as high flow, CPAP, vent mask, Non-rebreather mask): ……. Liters

|  | Upon admission | Worst values |
| --- | --- | --- |
| PO2 |  |  |
| pH |  |  |
| PCO2 |  |  |
| O2 saturation |  |  |

| **Treatment during hospitalization** | | |
| --- | --- | --- |
| Name of the medication | Strength | Duration of treatment |
|  |  |  |
|  |  |  |
|  |  |  |
